# Effects of Sub-Micro Sized BaTiO_3_ Blocking Particles and Ag-Deposited Nano-Sized BaTiO_3_ Hybrid Particles on Dielectric Properties of Poly(vinylidene-fluoride) Polymer

**DOI:** 10.3390/polym13213641

**Published:** 2021-10-22

**Authors:** Kanyapak Silakaew, Prasit Thongbai

**Affiliations:** 1 Materials Science and Nanotechnology Program, Faculty of Science, Khon Kaen University, Khon Kaen 40002, Thailand; kanyapak.slk@gmail.com; 2 Giant Dielectric and Computational Design Research Group (GD-CDR), Department of Physics, Faculty of Science, Khon Kaen University, Khon Kaen 40002, Thailand

**Keywords:** dielectric polymer composites, hybrid nanoparticles, poly(vinylidene fluoride), barium titanate, silver nanoparticle

## Abstract

This work provided an alternative route to balance the significantly increased dielectric permittivity (ε′) and effectively retained tanδ using an effective two-step concept. Ag-deposited nano-sized BaTiO_3_ (Ag-*n*BT) hybrid particle was used as the first filler to increase the ε′ of the poly(vinylidene-fluoride) (PVDF) polymer via the strong interfacial polarization and a high permittivity of *n*BT and suppress the increased loss tangent (tanδ) owing to the discrete growth of Ag nanoparticles on the surface of *n*BT, preventing a continuous percolating path. The ε′ and tanδ values at 10^3^ Hz of the Ag-*n*BT/PVDF composite with *f*_Ag-*n*BT_~0.29 were 61.7 and 0.036. The sub-micron-sized BaTiO_3_ (*μ*BT) particle was selected as the blocking particles to doubly reduce the tanδ with simultaneously enhanced ε′ due to the presence of the tetragonal BT phase. The *μ*BT blocking particles can effectively further inhibit the formation of conducting network and hence further reducing tanδ. By incorporation of *μ*BT clocking particles with *f_μ_*_BT_ = 0.2, the ε′ value of the Ag-*n*BT/PVDF-*μ*BT composite (*f*_Ag-*n*BT_ = 0.30) can significantly increase to 161.4, while the tanδ was reduced to 0.026. Furthermore, the tanδ was lower than 0.09 in the temperature range of −60–150 °C due to the blocking effect of *μ*BT particles.

## 1. Introduction

Recently, poly(vinylidene-fluoride) (PVDF) polymer, a dielectric material, has received much attention in electronic devices. Since PVDF has a low dielectric constant (ε′~10) compared to those of dielectric oxides [1,2], many research groups have intensively studied the improvement of the dielectric properties of the PVDF polymer for use in various applications (e.g., capacitors [3], actuators [4], and transducers [5]). An outstanding method to obtain better dielectric properties is to fabricate PVDF polymer composites. Dielectric oxide/PVDF composites are extensively studied, such as BaTiO_3_ (BT)/PVDF [6], SrTiO_3_/PVDF [7], Na_1/3_Ca_1/3_Bi_1/3_Ti_4_O_15_/PVDF [8,9], La_1.5_Sr_0.5_NiO_4_/PVDF [10], TiO_2_ nanorod/PVDF [11], and CaCu_3_Ti_4_O_12_ CCTO)/PVDF [12]. The ε′ value can be increased in all composite systems. However, the ε′ values of many dielectric oxide/PVDF composite systems were still lower than 10^2^ even when the volume fraction (*f*) was 0.5. Furthermore, the tanδ values of these composites were very large (>0.1) at *f* = 0.5. According to our previous work [13], the high ε′~66.1 (10^3^ Hz) and tanδ ~ 0.218 were achieved in the nano-sized BT (*n*BT)/PVDF nanocomposite with *f_n_*_BT_ = 0.4. Moreover, a low-frequency tanδ value is usually high due to polarization relaxation [6].

In addition to dielectric oxides, metal nanoparticles and conductive carbons have widely been used as a filler in the PVDF and other polymer composites such as Ni@NiO/PVDF [14], Al/PVDF [15], Zn/PVDF [16], Ag/PVDF [17], and multiwalled carbon nanotubes/PE (or epoxy) [18,19]. A rapidly enhanced ε′ of metal/PVDF composites can be accomplished using a low content of metal particles. The rapidly increased ε′ is related to the percolation theory. Although the ε′ values of many metal/PVDF composite systems can be significantly increased, a rapidly increased tanδ value (>>1.0) is usually obtained near a percolation threshold [20].

From the above limitations, a novel polymer composite system has been fabricated. Dielectric oxide and metal particles were simultaneously used as fillers in the PVDF polymer matrix, i.e., a 3-phase polymer composite [21,22,23,24,25,26,27,28,29]. As well known, the surface modification of a filler can be used to homogeneously disperse filler particles in polymer nanocomposites [8,23,30]. The ε′ values of ceramic/metal/PVDF composites can be increased. At 10^3^ Hz, the ε′ value of the Ag-BT/PVDF composite with at *f*_Ag-BT_ = 0.568 (hereafter referred to as the (0.568)[Ag-BT]/PVDF) was ~160, while the increase in tanδ was suppressed at the level of ~0.11 [22]. The ε′ and tanδ values of the (0.5)Ba(Fe_0.5_Nb _0.5_)O_3_/(0.25)Ni/PVDF were 475 and 0.61 at 10^2^ Hz [31]. The ε′ values of the 3-phase polymer composites are usually much larger than 100. According to the previous work [32], we studied the effect of additional blocking nanoparticles into 3-phase polymer composites. The Ag deposited on sub-micron-sized BT (*μ*BT)/nBT incorporated PVDF composites. A high ε′ value (ε′ = 165.2) with low tanδ value of ≈ 0.087 was obtained in the (0.51)[Ag-*μ*BT]/PVDF-(0.2)*n*BT composite. The obtained tanδ value was lower than that of the (0.568)[Ag-BT]/PVDF) composite [22]. Therefore, the blocking particles can suppress the tanδ value of 3-phase polymer composites. However, the tanδ value of the (0.51)[Ag-*μ*BT]/PVDF-(0.2)*n*BT composite was still quite high (>0.05), which can cause energy dissipation when it was used as a capacitor. There are some disadvantages found in the Ag-*μ*BT/PVDF-0.2*n*BT composites. First, a low surface area of the *μ*BT particles, which were used as the primary filler deposited by Ag, giving rise to the low content of the deposited Ag nanoparticles. Second, the *n*BT particles were too small for effectively blocking the conduction pathway and inhibiting the agglomeration of Ag-*μ*BT hybrid particles. These disadvantages must be resolved to achieve the maximized properties using the blocking concept. According to our previous report [33], we found that the *μ*BT particles could disperse multiwall carbon nanotube (MWCNT) in the PVDF polymer matrix better than that of *n*BT particles, giving rise to enhanced dielectric properties of the *μ*BT/MWCNT/PVDF composites.

It is expected that the optimizations of the dielectric properties with further improved dielectric properties may be achieved using Ag-*n*BT hybrid particles with large surface areas and by controlling their dispersion using *μ*BT particles. Thus, this work aims to systematically enhance the dielectric properties of the PVDF polymer using Ag-*n*BT hybrid particles as the primary filler to increase the ε′ and suppress the increased tanδ, and *μ*BT as the sub-micron blocking particles for effectively inhibiting the conducting network between Ag-*n*BT hybrid nanoparticles.

In this work, *n*BT was used to fabricate a hybrid particle due to its high surface area, while *μ*BT was used to improve blocking efficiency. The Ag-*n*BT/PVDF-*μ*BT polymer composites with different volume fractions of Ag-*n*BT (*f*_Ag-*n*BT_) were fabricated. The volume fraction of *μ*BT blocking particles was fixed at 0.2. The results revealed a significant increase in ε′ of Ag-*n*BT/PVDF-*μ*BT polymer composites. Surprisingly, the tanδ values of the polymer composites were very low for all compositions, while the ε′ increased with increasing *f*_Ag-*n*BT_. The relevant mechanisms were discussed in detail.

## 2. Experimental Details

### 2.1. Synthesis of Ag-nBT Hybrid Particles

**Figure 1:**
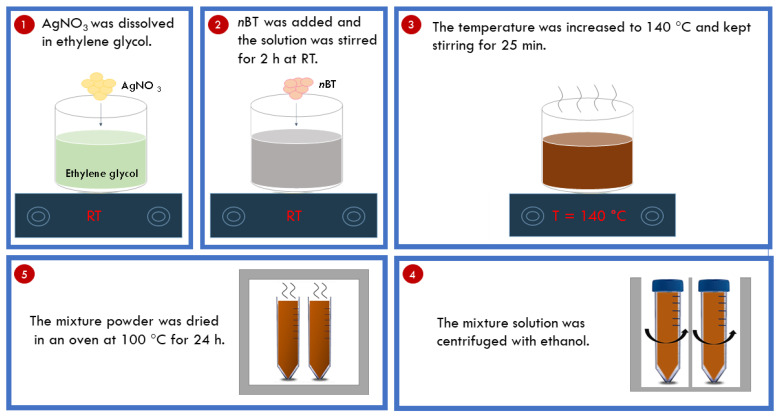
Schematic figure of the synthesis of Ag-*n*BT hybrid particles.

The raw materials for the synthesis of Ag-*n*BT hybrid particles consisted of BT (Sigma-Aldrich (St. Louis, MO, USA)) with a particle size of <100 nm and silver nitrate (AgNO_3_) (RCI Labscan, 99.8% purity). First, 5 g of AgNO_3_ was dissolved in 300 mL of ethylene glycol, and 5 g of *n*BT was added to the solution later. The mixture solution was stirred for 2 h at room temperature (*RT*). Secondly, the temperature increased to 140 °C and kept stirring for 25 min. Next, the mixture solution was centrifuged with ethanol to obtain the mixture powder. Finally, the mixture powder was dried in an oven at 100 °C for 24 h. A schematic figure of the synthesis of Ag-*n*BT hybrid particles is demonstrated in Figure 1.

### 2.2. Synthesis of Ag-nBT/PVDF-μBT Composites

The BT with a particle size of <1 μm (*μ*BT) (Sigma-Aldrich) was used as blocking particles to prevent the formation of conducting network between Ag-*n*BT hybrid particles. Therefore, PVDF-*μ*BT was considered a matrix in this composites system. A homemade ball-milling machine was used to mix the starting powders, consisting of two rotating horizontal axles and a rotating horizontal polyethylene jar that is partly filled with ZrO_2_ balls with 2.0 mm in diameter and particles to be mixed. The volume fraction ratio of ZrO_2_ balls to total starting powders and ethanol was 0.4:0.4. First, the PVDF and *μ*BT particles with a volume fraction ratio of 0.8:0.2 were mixed by ball-milling method in ethanol for 3 h. The speed of rotation was ~150 rpm. Second, the mixture was dried at 100 °C for 24 h to remove ethanol. Next, the mixed PVDF-*μ*BT powder was further mixed with Ag-*n*BT hybrid particles using a ball-milling method in ethanol. Then, ethanol was evaporated. Finally, the polymer composites powder of each volume fraction was molded by the hot-pressing method at 200 °C for 0.5 h. The nanocomposite disks with a thickness of ~0.6–1.0 mm and a diameter of ~12 mm were achieved.

### 2.3. Characterization Techniques

Transmission electron microscopy (Eindhoven, The Netherlands) (TEM, FEI, TECNAI G^2^ 20) was used to reveal the hybrid particles. X-ray diffractometry (Almelo, The Netherlands) (XRD, PANalytical, EMPYREAN) technique was used to examine the phase composition of the hybrid particles and composites. The microstructure of the polymer composites was displayed using Field Emission Scanning Electron Microscopy (Hillsboro, OR, USA) (FESEM, FEI, Helios NanoLab G3 CX) with an energy dispersive X-ray spectrometer (EDS). Fourier transformed infrared spectroscopy (FTIR, Bruker, TENSOR27) was used to indicate the phase conformation of the polymer composites. Dielectric properties were measured at a frequency range of 10^2^–10^6^ Hz, a temperature range of −60–150 °C and 500 mV of oscillation voltage using KEYSIGHT E4990A Impedance Analyzer (Santa Rosa, CA, USA).

## 3. Results and Discussion

**Figure 2:**
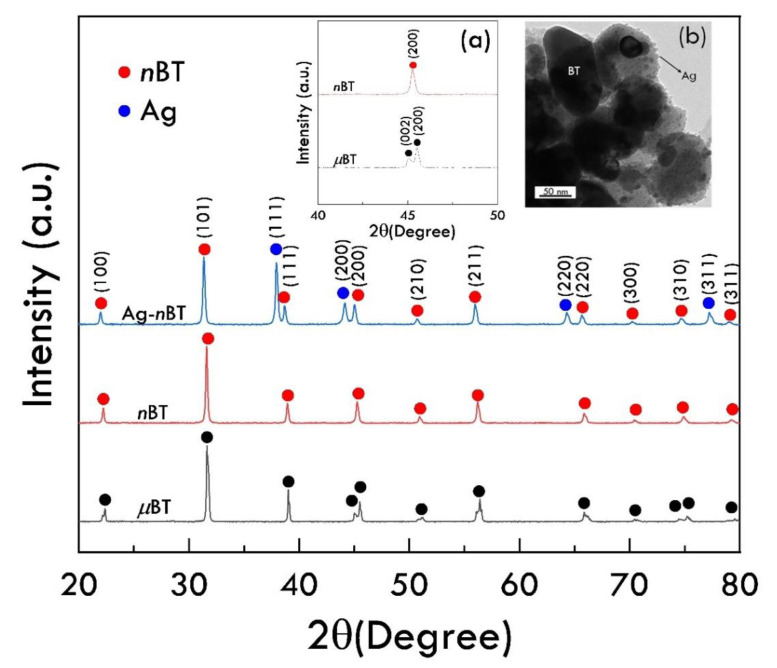
XRD patterns of *μ*BT, *n*BT, and Ag-*n*BT hybrid particles; insets (**a**) and (**b**) show expanded view near 2θ ≈ 45°, revealing the tetragonal phase of BT, and TEM image of hybrid particles, respectively.

Figure 2 shows the XRD pattern of the *μ*BT, *n*BT, and Ag-*n*BT hybrid particles, confirming the presence of BT and Ag phases. Both BT and Ag phases can be observed in the XRD pattern of Ag-*n*BT hybrid particles. Usually, the tetragonal phase structure of BT ceramics is observed below the curie temperature (~120 °C). As shown in inset (a), the characteristic peak at 2θ ≈ 45° ((200) plane) of the *n*BT appears a single peak, indicating a cubic perovskite structure in the ABO_3_ family for the *n*BT particles used [6,34]. On the other hand, double peaks at 2θ ≈ 45° of the tetragonality structure were observed in the XRD pattern of *μ*BT. It is expected that the ε′ value of the *μ*BT particle is larger than that of the *n*BT owing to the ferroelectric phase in the *μ*BT particle. The Ag diffraction planes (111), (200), (270), and (311) are located at 2θ around 38°, 44°, 65° and 78°, respectively [22,35]. The Ag-*n*BT hybrid particles have been successfully synthesized, and the phase structure of the *n*BT was unchanged. The inset (b) demonstrates the morphologies of the Ag-*n*BT hybrid particles, which were studied using the TEM technique. The TEM image shows the discretely deposited Ag particles on *n*BT surface. The particle sizes of the *n*BT and Ag nanoparticles are around 50–100 and <10 nm, respectively.

**Figure 3:**
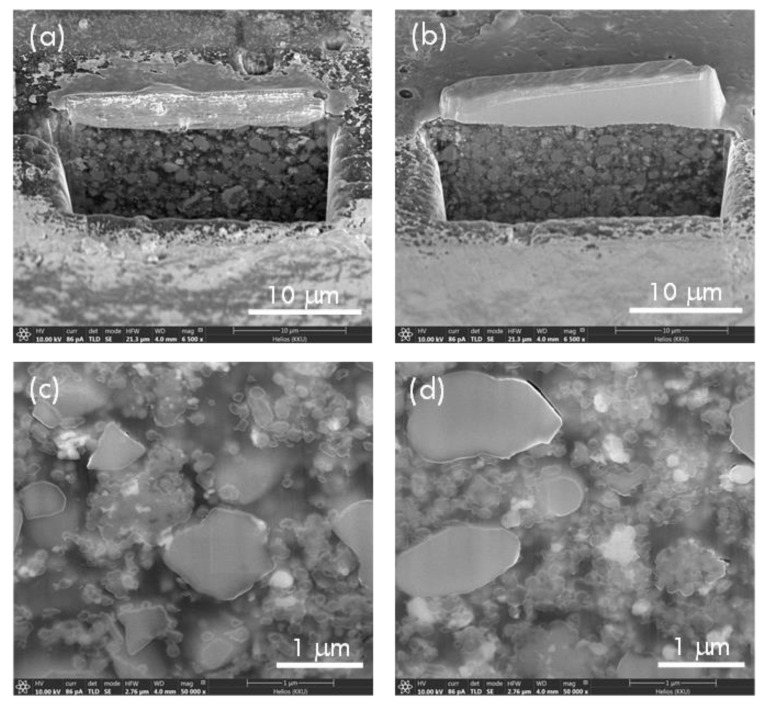
Cross-section images of the Ag-*n*BT/PVDF-(0.2)*μ*BT composites with (**a**,**c**) *f*_Ag-nBT_ = 0.25 and (**b**,**d**) *f*_Ag-nBT_ = 0.30.

The FESEM technique was used to reveal the microstructure of the Ag-*n*BT/PVDF-*μ*BT polymer composites. The outer surface of the composites was removed by focus ion, as shown in Figure 3a,b. The inner cores of the (0.25)[Ag-*n*BT]/PVDF-(0.2)*μ*BT and (0.3)[Ag-*n*BT]/PVDF-(0.2)*μ*BT composites are revealed using FESEM, as shown in Figure 3c,d, respectively. showing the dispersion of *μ*BT and Ag-*n*BT hybrid particles. The agglomeration of the *μ*BT blocking particles disappeared. The *μ*BT particles were well dispersed in the polymer matrix. As a result, the conducting network of Ag-*n*BT hybrid particles can be inhibited, which may lead to a reduction in tanδ values and conductivity of the polymer composites.

**Figure 4:**
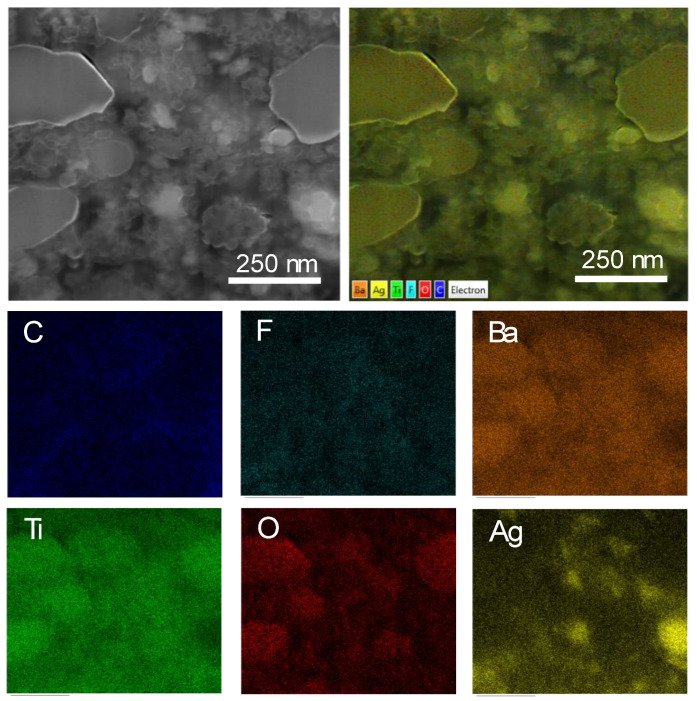
SEM-EDX elemental mapping of the Ag-*n*BT/PVDF-(0.2)*μ*BT composites with *f*_Ag-nBT_ = 0.30.

The SEM-EDS elemental mapping technique was used to show the dispersion of fillers, especially for the Ag nanoparticles, in the polymer composites. Figure 4 shows the SEM-EDX elemental mapping of all elements in the (0.3)[Ag-*n*BT]/PVDF-(0.2)*μ*BT composite. Barium (Ba), Titanium (Ti), and Oxygen (O), which are the elements of BT particles, were distributed throughout the PVDF matrix. Moreover, the elemental mapping of Ag nanoparticles was also revealed. There were some small clusters of Ag nanoparticles, which can be observed as a bright spot in the SEM-EDS mapping image of Ag. However, the clusters of Ag particles were not enough to create the conductive pathways. The Ag element disappeared in the *μ*BT areas. The presence of all elements for BT and Ag particles confirmed that the PVDF matrix was filled with the Ag-*n*BT hybrid particles and *μ*BT particles. In addition, Carbon (C) and Fluorine (F) represented the composition of the PVDF polymer. It is worth noting that for practical application in capacitors, the dielectric layer was sandwiched by two metal electrodes. The electrode and dielectric layers are usually encapsulated by an insulating polymer to prevent the hydration of a dielectric layer. Furthermore, the perovskite-BT particles were embedded in the PVDF polymer matrix; thus, the effect of humidity on the dielectric properties of BT was double protected.

**Figure 5:**
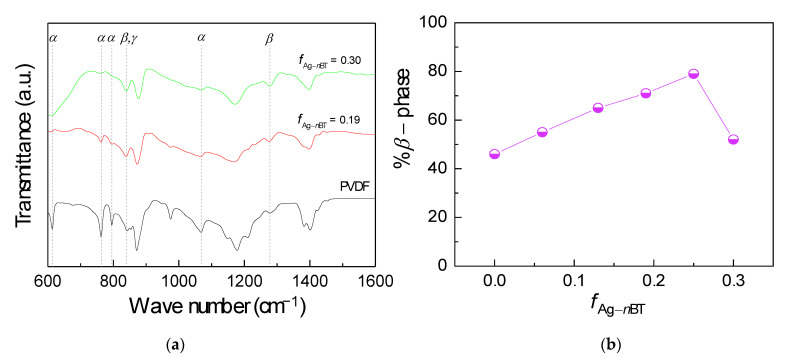
(**a**) FTIR spectra of PVDF and the Ag-*n*BT/PVDF-(0.2)*μ*BT composites and (**b**) % *β*-phase of the Ag-*n*BT/PVDF-(0.2)*μ*BT composites with various *f*_Ag-nBT_.

The phase conformation of the PVDF matrix in the polymer composites was restudied again with FTIR spectroscopy. As displayed in Figure 5a, the *α*-phase located at wavenumber around 614, 766, 795, and 976 cm^−1^ [36,37,38]. Furthermore, the peak at wavenumber about 840 cm^−1^ was representative of both the *γ*- and *β*-phases [37,38]. The *β*-phase also appeared at wavenumber around 1279 cm^−1^ [36,37]. There were *α-, γ-* and *β-*phases in the PVDF polymer and polymer composites. As well known, the *β*-phase has the highest polarity among all phase conformation of PVDF [37]. The *β*-phase can affect the dielectric properties of the PVDF polymer composites. The Lambert-Beer equation was used to calculate the content of *β-*phase (*F*(*β*)), assuming that there are only *α- and β-*phases in the polymer composites [37]. The equation can be presented as follows,

(1) $$F(\beta )=\frac{A_{\beta }}{(K_{\beta }/K_{\alpha })A_{\alpha }+A_{\beta }}\;,\;$$

where *A_α_* and *A_β_* represent the absorbance of *α*-, *β*-phases at wavenumber = 766 cm^−1^ and 840 cm^−1^, respectively. *K_α_* (6.1 × 10^4^ cm^2^mol^−1^) is the absorbance coefficient of *α*-phase. *K_β_* = 7.7 × 10^4^ (cm^2^mol^−1^) is the absorbance coefficient of *β*-phases. The calculated values of *F*(*β*) are displayed in Figure 5b. The *F*(*β*) values of the PVDF and the Ag-*n*BT/PVDF-(0.2)*μ*BT composites with *f*_Ag-*n*BT_ = 0.06, 0.13, 0.19, 0.25 and 0.30 were 46, 55, 65, 71, 79, and 52% for the polymer composites, respectively. The *F*(*β*) increased with increasing *f*_Ag-*n*BT_ from 0.06 to 0.25, which was attributed to the negatively charged of filler induced the formation of *β*-phase (all trans, TTT) [37]. However, the *β*-phase conformation was inhibited in the case of *f*_Ag-*n*BT_ = 0.3, leading to low value of *F*(*β*) [39].

**Figure 6:**
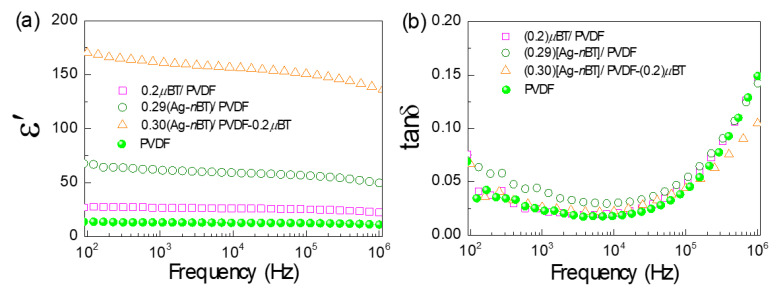
Comparison of (**a**) ε′ and (**b**) tanδ at *RT* for PVDF polymer, (0.2)*μ*BT/PVDF, (0.29)[Ag-*n*BT]/PVDF, and (0.30)Ag-*n*BT/PVDF-(0.2)*μ*BT composites.

The dielectric properties at *RT* in the frequency range of 10^2^–10^6^ Hz of the PVDF polymer, 2-phase (0.2)*μ*BT/PVDF (or PVDF-(0.2)*μ*BT), 3-phase (0.29)[Ag-*n*BT]/PVDF and (0.30)[Ag-*n*BT]/PVDF-(0.2)*μ*BT composites are demonstrated in Figure 6. Please note that it is difficult to assign the same *f*_Ag-*n*BT_ of the Ag-*n*BT/PVDF and Ag-*n*BT/PVDF-(0.2)*μ*BT composites in the experimental process. Nevertheless, significantly different dielectric properties between the (0.29)[Ag-*n*BT]/PVDF and (0.30)[Ag-*n*BT]/PVDF-(0.2)*μ*BT composites can be observed while the *f*_Ag-*n*BT_ values of these two composites are different slightly. At 10^3^ Hz, the ε′ and tanδ values of the PVDF polymer are 13.0 and 0.022, respectively. The ε′ and tanδ values of the (0.2)*μ*BT/PVDF composite are 26.7 and 0.022, respectively. These values are similar to those reported in the previous works for the PVDF polymer composite filled with *μ*BT particles [6]. This result shows the important role of the *μ*BT particles to enhance the dielectric response in the PVDF polymer without any effect on the tanδ value. The ε′ and tanδ values at 10^3^ Hz of the (0.29)[Ag-*n*BT]/PVDF composite without blocking particles are 61.7 and 0.036, respectively. The tanδ of the (0.29)[Ag-*n*BT]/PVDF composite was slightly higher than those of the PVDF polymer and (0.2)*μ*BT/PVDF composite over the measured frequency range, whereas the ε′ of the (0.29)[Ag-*n*BT]/PVDF composite was much larger. This result indicated that the ε′ of the PVDF polymer can be significantly increased by incorporating with Ag-*n*BT hybrid particles due to the strong interfacial polarization (i.e., Ag-*n*BT and Ag-PVDF interfaces) and a relatively high permittivity of *n*BT particles compared to that of the PVDF polymer.

Generally, the tanδ of PVDF polymer composites filled with conductive nanoparticles is largely increased as the filler loading increases. The suppressed tanδ in the (0.29)[Ag-*n*BT]/PVDF composite was owing to the discrete growth of Ag nanoparticles on the surface of *n*BT, preventing the direct contact between Ag nanoparticles. The ε′ and tanδ values at 10^3^ Hz of the (0.3)[Ag-*n*BT]/PVDF-(0.2)*μ*BT composite with blocking particles are 161 and 0.026, respectively. Obviously, the *μ*BT blocking particles can further improve the dielectric properties of the (0.30)[Ag-*n*BT]/PVDF-(0.2)*μ*BT composite, resulting in a further significantly increased ε′ with simultaneously reducing the tanδ to the initial value of the PVDF polymer. As shown in Figure 6a, the ε′ of the (0.30)[Ag-*n*BT]/PVDF-(0.2)*μ*BT composite increased by factors of 6 and 12 compared to that of the (0.2)*μ*BT/PVDF composite and PVDF polymer, respectively, while the tanδ values of these three samples are nearly the same in value. This result indicated the essential role of Ag-*n*BT hybrid particles for increasing the dielectric response without any effect on the tanδ. The important role of the *μ*BT blocking particles is to efficiently obstruct the conductive pathways with simultaneously increasing the ε′ due to the tetragonal ferroelectric phase of the *μ*BT. Therefore, the *μ*BT blocking particles were the key factor in low tanδ values of the Ag-*n*BT/PVDF-(0.2)*μ*BT composites. Typically, polymer nanocomposites can provide a high tanδ values. This is because of high surface energy, which may contribute to the agglomeration of nanoparticles [13]. Therefore, the addition of *μ*BT can also inhibit the agglomeration of the Ag-*n*BT hybrid particles. The ε′ and tanδ values are better than our previous work [32], which PVDF incorporated with the Ag-*μ*BT hybrid particles and *n*BT blocking particles.

**Figure 7:**
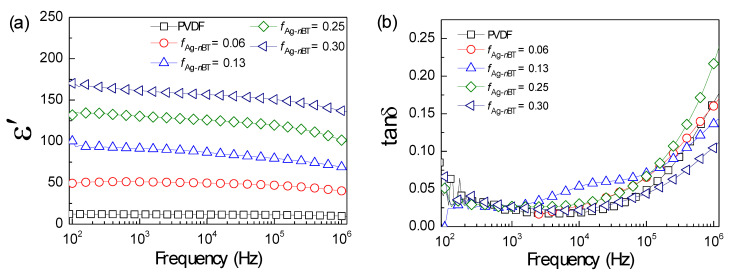
Frequency dependence on (**a**) ε′ and (**b**) tanδ of the Ag-*n*BT/PVDF-(0.2)*μ*BT composites with various *f*_Ag-nBT_.

The frequency-dependence behavior of dielectric properties of the Ag-*n*BT/PVDF-(0.2)*μ*BT composites with different *f*_Ag-*n*BT_ is displayed in Figure 7a. The ε′ increases with increasing *f*_Ag-*n*BT_ in the frequency range of 10^2^–10^6^ Hz, which is attributed to the significantly increased interfacial polarization and the increase in a high-permittivity *n*BT phase. Moreover, the ε′ is nearly independent of the frequency. However, the ε′ drops at a frequency range of 10^6^ Hz, which is noticeable at high *f*_Ag-*n*BT_. The frequency-dependence behavior of the ε′ value in a high-frequency range is usually ascribed by the dielectric relaxation of the PVDF matrix. The dipole polarization relaxation of the PVDF matrix was prominent at this frequency range [6]. Figure 7b demonstrates the variation in the tanδ in the frequency of 10^2^–10^6^ Hz. The low-frequency tanδ values of all composites are lower than 0.09. Notably, the tanδ value of the (0.30)[Ag-*n*BT]/PVDF-(0.2)*μ*BT is lower than 0.1 over the measured frequency range. In a high-frequency range, the increased tanδ value is usually due to the dielectric relaxation of the PVDF matrix, corresponding to the decreased ε′ value at high frequencies.

**Figure 8:**
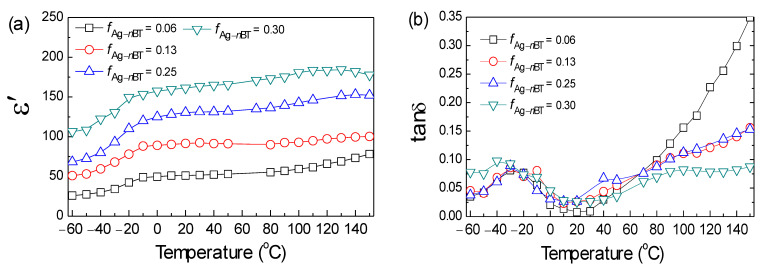
Temperature dependence on dielectric properties (**a**) ε′ and (**b**) tanδ of the Ag-*n*BT/PVDF-(0.2)*μ*BT composites with different *f*_Ag-nBT_.

The effects of temperature on the dielectric properties of the Ag-*n*BT/PVDF-(0.2)*μ*BT composites were also investigated. Figure 8a,b show the ε′ and tanδ values (at 10^3^ Hz) of the Ag-*n*BT/PVDF-(0.2)*μ*BT composites with various *f*_Ag-*n*BT_ in the temperature range of −60–150 °C, respectively. The rapid increase in ε′ is observed when the temperature increased from −60 to 0 °C. Then, it slightly increased as the temperature increased from 0 to 150 °C. The rapid increase in ε′ in a low-temperature range correlates to the *β* relaxation of the PVDF polymer [22,40,41]. The *β* relaxation corresponds to the dipolar group motions and the glass transition [40,41]. Furthermore, the ε′ at high temperatures is dominant by the interfacial polarization and molecular motions of PVDF [22,41,42]. The relaxation peak of tanδ in a low-temperature range is related to *β* relaxation, corresponding to the observed rapid change in the ε′. The tanδ value increases with increasing the temperature. The tanδ value in a high-temperature range of the composites with high *f*_Ag-*n*BT_ is lower than that of the composites with low *f*_Ag-*n*BT_, which is because the high fillers inhibited the movement of the PVDF chains, resulting in the lower tanδ [43]. 

## 4. Conclusions

The PVDF polymer composites filled with the Ag-*n*BT hybrid and *μ*BT particles were fabricated as the 3-phase Ag-*n*BT/PVDF-(0.2)*μ*BT composites. The *μ*BT particles were used as blocking particles to inhibit the formation of conducting network. The microstructure analysis revealed a homogeneous distribution of fillers in the PVDF matrix. The significantly improved dielectric properties of the Ag-*n*BT/PVDF-(0.2)*μ*BT composites were obtained. The ε′ values increased from 51.1 to 161.4, while the tanδ remained at a low value of <0.03. It was demonstrated that the addition of Ag-*n*BT hybrid particles could cause an increase in the ε′ of the polymer composites through interfacial polarization between filler-matrix and filler-filler. Moreover, the observed low tanδ and σ values indicated that there was no formation of the conducting network in the insulative PVDF polymer matrix. The *μ*BT particles played an essential role in suppressing the formation of the conductive pathways. Therefore, the results of microstructure, dielectric properties, including electrical properties indicated that the Ag-*n*BT/PVDF-(0.2)*μ*BT composites is a promising dielectric polymer composite, which has a potential for application in in electronic devices.

## Data Availability

The data presented in this study are available on request from the corresponding author.
